# Metabolites of Cerebellar Neurons and Hippocampal Neurons Play Opposite Roles in Pathogenesis of Alzheimer's Disease

**DOI:** 10.1371/journal.pone.0005530

**Published:** 2009-05-13

**Authors:** Jing Du, Bing Sun, Kui Chen, Lang Zhang, Shubo Liu, Qingquan Gu, Li Fan, Nanming Zhao, Zhao Wang

**Affiliations:** 1 Protein Science Key Laboratory of the Ministry of Education, Department of Biological Sciences and Biotechnology, School of Medicine, Tsinghua University, Beijing, People's Republic of China; 2 Department of Pharmacology, Anhui Medical University, Hefei, Anhui, China; 3 Cardiovascular Research, Starr Academic Center, Providence Heart & Vascular Institute, Portland, Oregon, United States of America; University of North Dakota, United States of America

## Abstract

Metabolites of neural cells, is known to have a significant effect on the normal physiology and function of neurons in brain. However, whether they play a role in pathogenesis of neurodegenerative diseases is unknown. Here, we show that metabolites of neurons play essential role in the pathogenesis of Alzheimer's disease (AD). Firstly, *in vivo* and *in vitro* metabolites of cerebellar neurons both significantly induced the expression of Aβ-degrading enzymes in the hippocampus and cerebral cortex and promoted Aβ clearance. Moreover, metabolites of cerebellar neurons significantly reduced brain Aβ levels and reversed cognitive impairments and other AD-like phenotypes of APP/PS1 transgenic mice, in both early and late stages of AD pathology. On the other hand, metabolites of hippocampal neurons reduced the expression of Aβ-degrading enzymes in the cerebellum and caused cerebellar neurodegeneration in APP/PS1 transgenic mice. Thus, we report, for the first time, that metabolites of neurons not only are required for maintaining the normal physiology of neurons but also play essential role in the pathogenesis of AD and may be responsible for the regional-specificity of Aβ deposition and AD pathology.

## Introduction

The fluid environment of neurons, which contains metabolites of neural cells, is known to have a significant effect on the normal physiology and function of neurons in brain [1]. For example, neurotrophic factors, which are secreted by target tissue and neural cells, can prevent the associated neurons from initiating programmed cell death and thereby allow them to survive [2], [3]. However, little is known about the role of metabolites of neurons in the pathogenesis of neurodegenerative diseases.

Alzheimer's disease (AD) is a progressive neurodegenerative disease and the major cause of dementia among seniors. There are more than 4 million people suffering from AD in the USA and 12 million worldwide. Ten percent of Americans over the age of 65 and half of those over 85 have AD [4], [5]. AD pathology is characterized by amyloid deposits in certain regions of the brain, such as the entorhinal cortex, hippocampus and basal forebrain. All of these areas are small, specialized structures in the brain that play critical roles in memory [6], [7]. However, the cerebellum is spared from significant amyloid-β (Aβ) accumulation and neurotoxicity induced by Aβ, even though Aβ is present throughout the brain [8]. Even in the AD-damaged regions, the pathology is not ubiquitous. For example, the CA1 and the SB regions of the hippocampus are vulnerable to AD, whereas the CA3 region is resistant to AD [9]. Neuronal loss in CA1 area of the hippocampus is demonstrated to be correlated with the duration and severity of AD [10], [11]. In cerebellum, Aβ does not deposit as senile plaques but as diffuse plaques composed of nonfibrillar Aβ [12]. It has also been reported that cerebellar neurons are more resistant to soluble oligomeric Aβ an Aβ species with potent neurotoxic activities [13], than the cortex and hippocampus, which are vulnerable to AD [14]. However, little is known about the physiological mechanism underlying the regional specificity of Aβ accumulation.

In the present study, we investigated whether the metabolites of neurons in their fluid environment contribute to the pathogenesis of AD.

## Results and Discussion

### Results

#### Metabolites of cerebellar neurons induced expression of Aβ degrading enzymes and promoted Aβ clearance in hippocampal neurons

**Figure 1:**
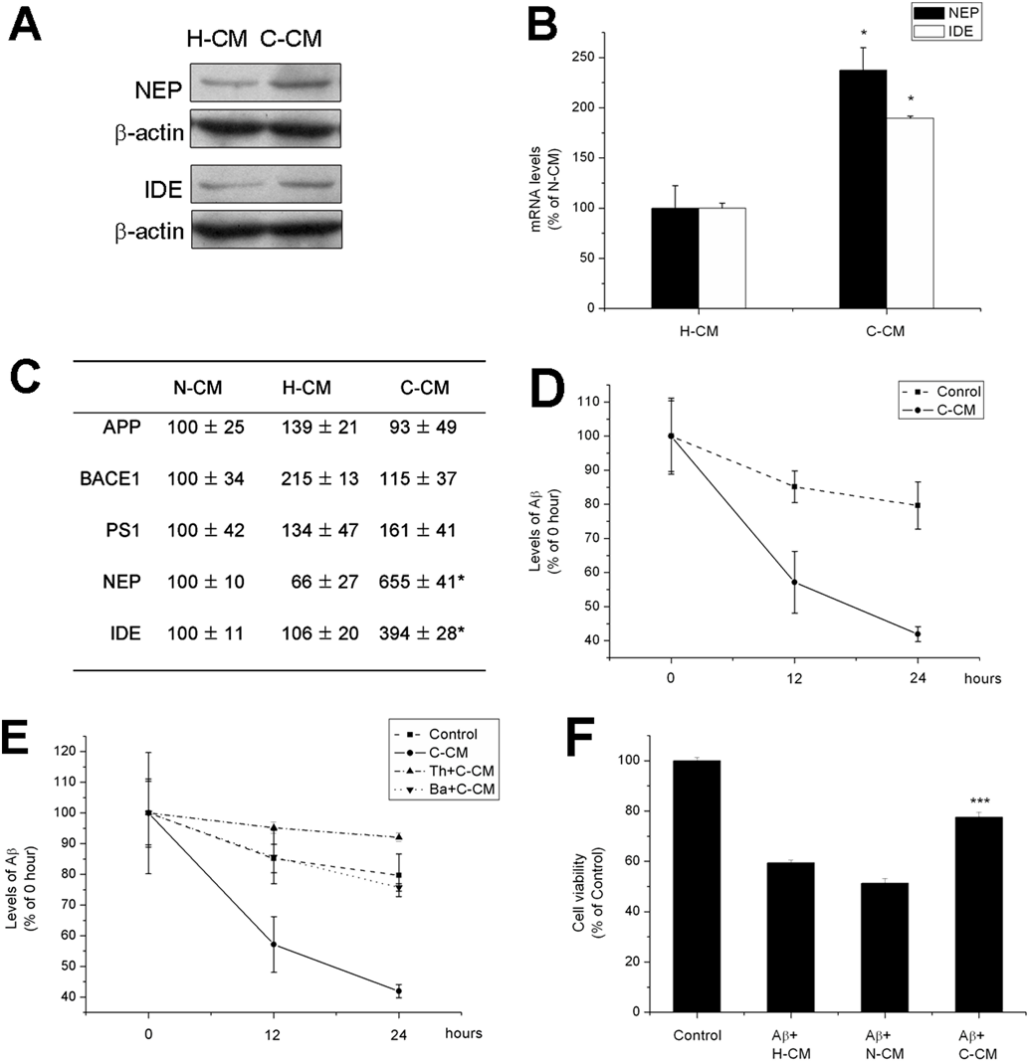
Metabolites of cerebellar neurons induced expression of Aβ degrading enzymes and promoted Aβ clearance in hippocampal neurons. (A) Western blot analysis of NEP and IDE expression in hippocampal neurons treated with conditioned medium. (B) Real-time PCR analysis of NEP and IDE expression in hippocampal neurons treated with conditioned medium (*P<0.05). (C) Real-time PCR analysis of gene expression in the hippocampus of SD rats treated with conditioned medium (*P<0.05). (D) The Aβ_1-40_ ELISA result shows that the decrease in Aβ (initial concentration of Aβ_1-40_ monomers was 2 µg/ml) was significantly accelerated by C-CM compared to H-CM (*P*<0.01). (E) The Aβ_1-40_ ELISA result shows that inhibitor of NEP (Thiorphan, Th; 10 µM) and IDE (Bacitracin, Ba; 0.5 mg/ml) attenuated the promotion of Aβ clearance by C-CM (*P* = 0.001 and *P*<0.0005, respectively). (F) Cell activity was optically measured as a reduction of the MTT dye uptake by viable cells after a 48 h exposure to Aβ in the presence of C-CM, H-CM or N-CM (*** *P*<0.005).

We first found that expression of Aβ degrading enzymes, neprilysin (NEP) and insulin degrading enzyme (IDE) in hippocampal neurons was induced by exposure to conditioned medium from cerebellar neurons (C-CM) , which contains metabolites of cerebellar neurons, compared to treatment with conditioned medium from hippocampal neurons (H-CM) containing hippocampal neuron metabolites (Figure 1A and B). Moreover, injection of concentrated C-CM to the lateral ventricle of SD rats also induced IDE and NEP expression (Figure 1C). Secondly, clearance of exogenous Aβ by primary hippocampal neurons was significantly facilitated by the addition of C-CM, compared to treatment with H-CM via Aβ degradation by IDE and NEP (Figure 1D and E). C-CM also protected hippocampal neurons from Aβ neurotoxicity compared to H-CM and fresh medium (N-CM), which does not contain any neuronal metabolite (Figure 1F). Similar results were obtained in primary cortical neurons as opposed to hippocampal neurons (data not shown). These results suggest that C-CM, which contains metabolites of cerebellar neurons, promoted Aβ clearance by inducing expression of Aβ degrading enzymes in hippocampal neurons and protected hippocampal neurons from Aβ neurotoxicity.

#### Metabolites of cerebellar neurons reduced Aβ levels in the hippocampus of APP/PS1 transgenic mice

**Figure 2:**
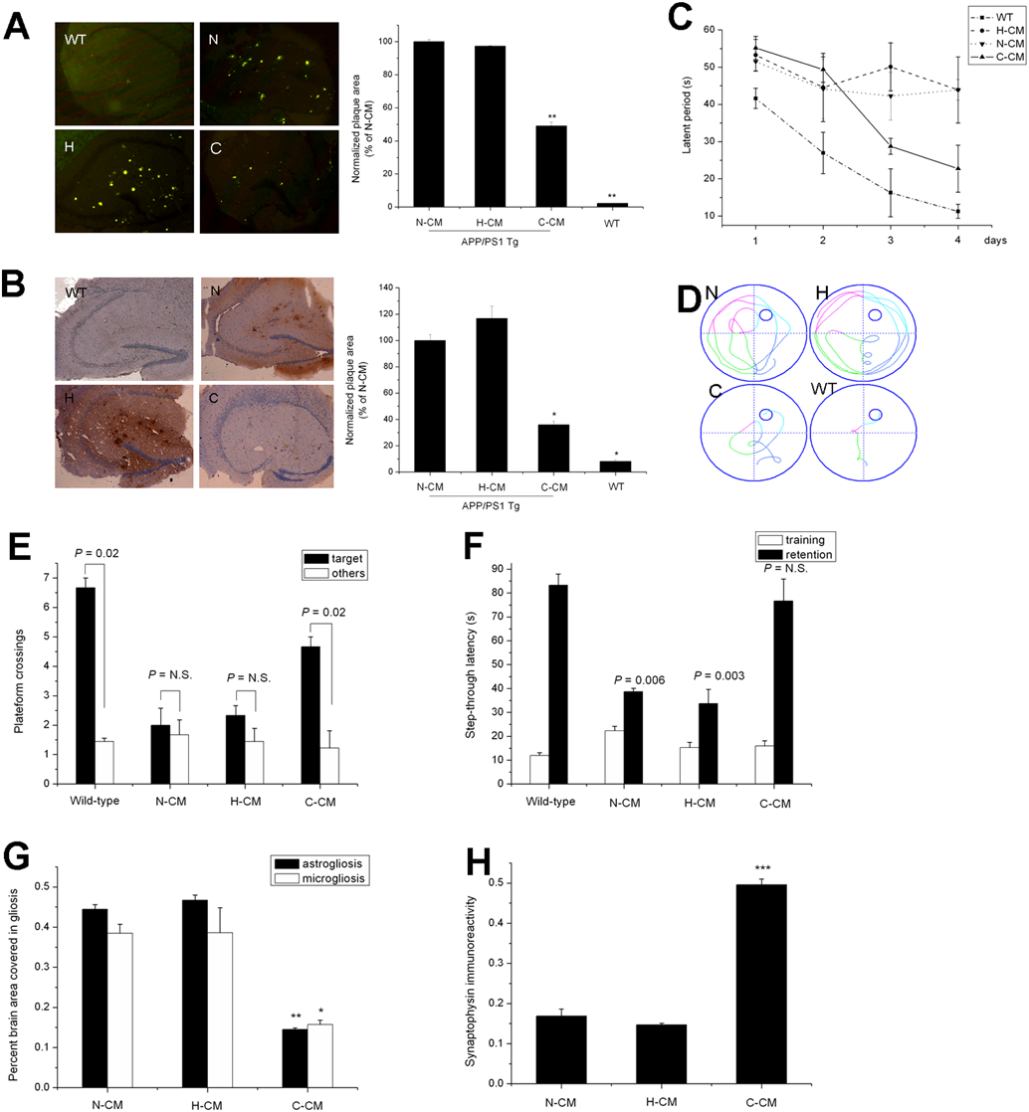
Metabolites of cerebellar neurons reversed Alzheimer disease-like phenotypes of APP/PS1 transgenic mice. (A) Thioflavin-S staining of hippocampal amyloid plaques in APP/PS1 mice (**P<0.01). (B) Immunostaining of hippocampal Aβ deposits in APP/PS1 mice (n = 6 to 10 mice per treatment group; age 12 months) (*P<0.05). (C) Cued platform learning curves show that APP/PS1 mice treated with C-CM had significant behavioral improvement compared to mice treated with either H-CM or N-CM (*P*<0.01). (D) Representative path tracings of probe trials after 4 days of training. (E) Number of target platform crossings versus crossings of the equivalent area in the three other quadrants. (F) Latencies to step through into the shock compartment on the training trial and retention trial of the passive avoidance test. (G) C-CM decreased both the astrogliotic response (**P<0.01) and microglial activation (*P<0.05). (H) C-CM increased the number of syanptophysin-reactive boutons and cell bodies compared to N-CM and H-CM (***P<0.005; n = 6 to 10 mice per treatment group; age 12 months).

Next, concentrated C-CM was injected into the lateral ventricle of APP/PS1 transgenic mice, which were known as AD models, to investigate its effect on endogenous Aβ levels. APP/PS1 mice injected with H-CM were used as negative controls and mice injected with concentrated N-CM were used as blank controls. The levels of both soluble and insoluble Aβ in the hippocampus of APP/PS1 mice injected with C-CM were markedly reduced two weeks after treatment compared to those in control mice. Because brain Aβ_1-42_ levels in APP/PS1 mice were much lower than Aβ_1-40_ levels, we only compared Aβ_1-40_ levels in the ELISA analysis. Moreover, metabolites of cerebellar neurons also reduced the numbers and mean sizes of senile plaques in the hippocampus of APP/PS1 transgenic mice (Table 1; Figure 2A and B). Similar results were recorded for cortical Aβ levels (Table S1). Our results demonstrate that metabolites of cerebellar neurons reduced brain Aβ levels in APP/PS1 transgenic mice. We also found that metabolites of rat cerebellar neurons significantly decreased steady state levels of Aβ in hippocampus of normal SD rats (data not shown), which suggests a consensus function of metabolites of cerebellar neurons.

**Table 1:** Metabolites of cerebellar neurons decrease Aβ levels and plaques in hippocampus.

	Aβ_1-40_ (ng/g wet brain±s.e.m)		Total plaque area (µm^2^)	Mean plaque size (µm^2^)
	Soluble	Insoluble		
N-CM	45,350±609	78,110±4258	351,749±4655	420±20
H-CM	45,781±1090	108,195±3354	342,236±1817	458±22
C-CM	24,338±905#	35,897±1449**	172,942±8206**	194±4*
12-month LV	47,800±480	107,670±2173	298,233±7653	323±13
12-month 4V	27,417±953#	35,718±1996#	127,261±3635#	176±5**
4-month LV	22,284±667	37,611±1172	26,689 ±1281	86±6
4-month 4V	15,206±849**	16,427±1504#	11,014±890**	45±7*

# *P*<0.001.

** *P*<0.01.

* *P*<0.05.

#### Metabolites of cerebellar neurons reversed Alzheimer's disease-like phenotypes of APP/PS1 transgenic mice

Next, we explored whether the reduction in brain Aβ levels by metabolites of cerebellar neurons could eventually improve the cognitive function of APP/PS1 transgenic mice. The Morris Water Maze (MWM) was used to test learning and memory. At 12 months of age, APP/PS1 mice were significantly impaired in cognitive function compared with age-matched wild-type mice. Treated with C-CM had significant behavioral improvement compared to APP/PS1 mice treated with either H-CM or N-CM (*P*<0.01; Figure 2C). Probe trials, in which the platform was removed and mice were given 1 min to explore the pool, confirmed the beneficial effect of C-CM (Figure 1D and E). Thus, C-CM ameliorates water maze learning and memory deficits.

A passive avoidance test was also performed to examine learning and memory. Inspection of the results indicated that retention latency was higher than training latency, which is indicative of memory formation, in all groups (Figure 2F). The retention latency in APP/PS1 mice treated with C-CM was significantly higher than that in APP/PS1 mice treated with either H-CM or N-CM (Figure 2F).

These behavioral improvements were accompanied by improvements in other Alzheimer's disease-like neuropathology including glial reaction and synaptic loss. C-CM decreased both the astrogliotic response (*P*<0.01) and microglial activation (*P*<0.05; Figure 2G), compared to N-CM and H-CM treatment. Synaptic loss presumably reflects the chronic synaptotoxic effects of Aβ oligomers. Measurement of synaptophysin levels in the hippocampus showed that C-CM increased the number of syanptophysin-reactive boutons and cell bodies compared to N-CM and H-CM (*P*<0.01; Figure 2H). These results show that metabolites of cerebellar neurons reversed the Alzheimer's disease-like phenotypes of APP/PS1 transgenic mice.

#### *In vivo* metabolites of cerebellar neural cells reversed Alzheimer's disease-like phenotypes of APP/PS1 transgenic mice

**Figure 3:**
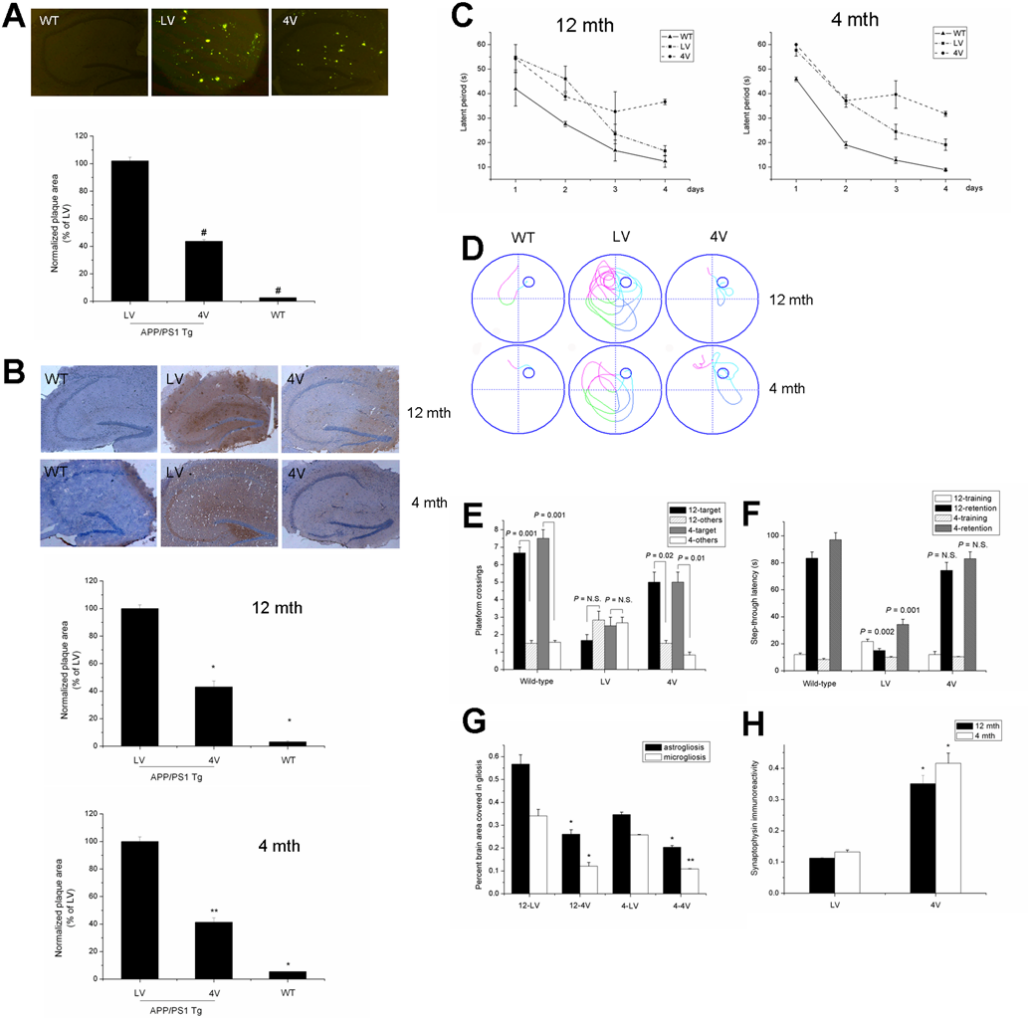
*In vivo* metabolites of cerebellar neurons reversed Alzheimer's disease-like phenotypes of APP/PS1 transgenic mice. (A) Thioflavin-S staining of hippocampal amyloid plaques in APP/PS1 mice (^#^ *P*<0.01). (B) Immunostaining of hippocampal Aβ deposits in APP/PS1 mice (*P<0.05, **P<0.01). (C) Cued platform learning curves showed that APP/PS1 mice treated with CSF-4V exhibited significant behavioral improvement compared to mice treated with CSF-LV (*P*<0.05). (D) Representative path tracings of probe trials after 4 days of training. (E) Number of target platform crossings versus crossings of the equivalent area in the three other quadrants. (F) Latencies to step through into the shock compartment on the training trial and retention trial of the passive avoidance test. (G) CSF-4V decreased both the astrogliotic response and microglial activation (*P<0.05, **P<0.01). (H) C-CM increased the number of syanptophysin-reactive boutons and cell bodies compared to N-CM and H-CM (*P<0.05; n = 6 to 10 mice per treatment arm; age 4 months or 12 months).

Since the cerebrospinal fluid of the fourth ventricle (CSF-4V) contains *in vivo* metabolites of cerebellar neural cells, we collected CSF-4V from wild-type mice and injected this into the lateral ventricle of APP/PS1 transgenic mice. CSF of the lateral ventricle (CSF-LV) was also collected and injected as a control. Injection of CSF-4V significantly reduced levels of both soluble and insoluble Aβ (Table 1; Figure 3A) and the numbers and mean sizes of senile plaques in the hippocampus of APP/PS1 transgenic mice (Figure 3B). Similar results were obtained in APP/PS1 mice at 12 months and 4 months, which are the late and early stages of AD pathology, respectively. We also found that CSF-4V from rat brains significantly decreased steady state levels of Aβ in hippocampus of normal SD rats (data not shown), which suggests a consensus function of CSF-4V. APP/PS1 mice, aged either 12 months or 4 months, exhibited significant memory improvement and better maze learning when they were treated with CSF-4V compared to mice treated with CSF-LV (*P*<0.05; Figure 3C). Probe trials, in which the platform was removed and mice were given 1 min to explore the pool, confirmed the beneficial effect of CSF-4V (Figure 3D and E). The retention latency of the passive avoidance test in APP/PS1 mice treated with CSF-4V was significantly higher than that in those treated with CSF-LV (Figure 3F).

The behavioral improvements after CSF-4V treatment were accompanied by improvements in other Alzheimer's disease-like neuropathology such as astrogliotic response, microglial activation (Figure 3G), and synaptic loss (Figure 3H), compared to CSF-LV. These results showed that *in vivo* metabolites of cerebellar neurons reversed Alzheimer's disease-like phenotypes of APP/PS1 transgenic mice in both early and late stages of AD pathology.

#### Metabolites of hippocampal neurons reduced Aβ-degrading enzymes expression and caused cerebellar neurodegeneration in APP/PS1 transgenic mice

**Figure 4:**
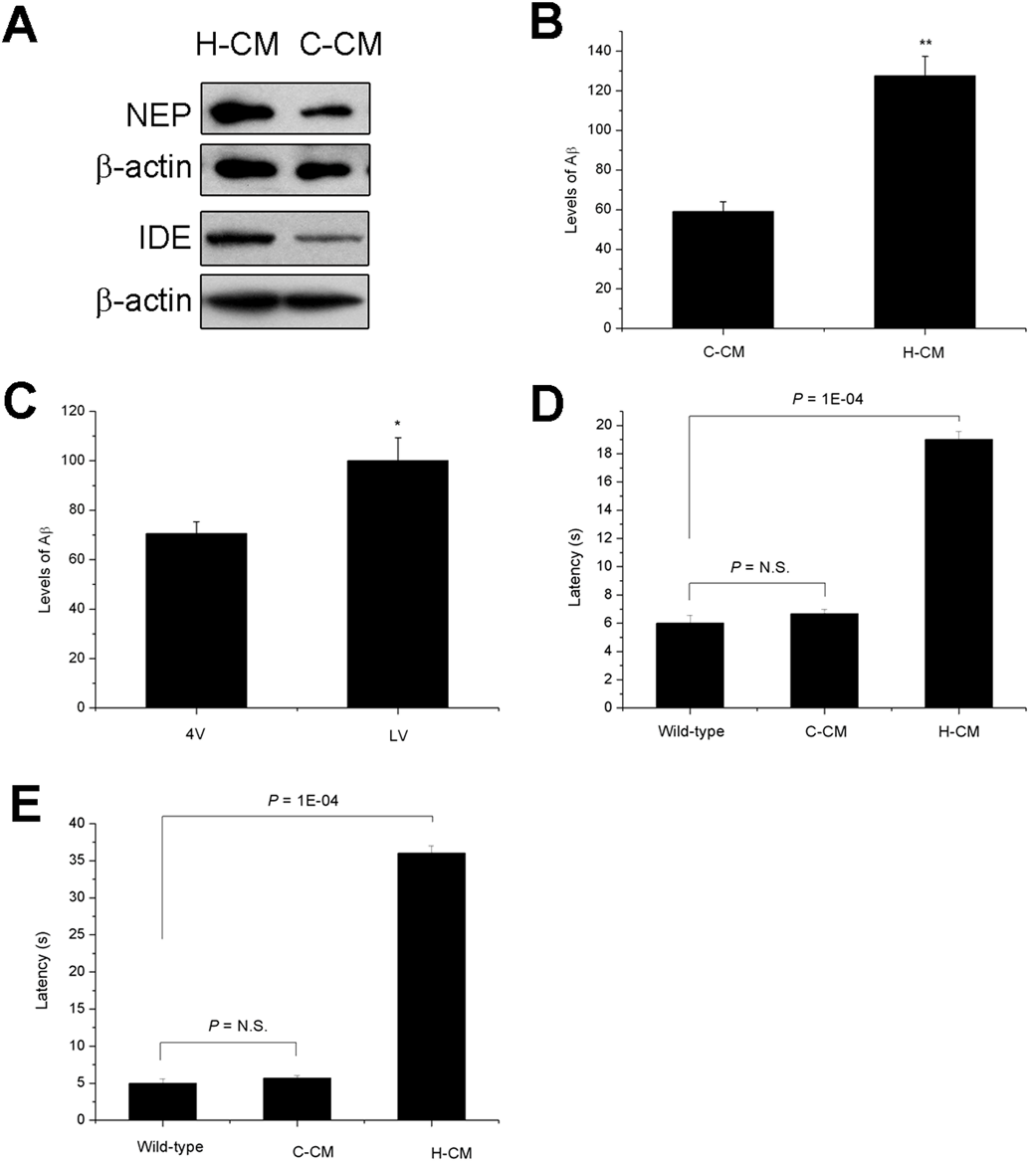
Metabolites of hippocampal neurons reduced Aβ-degrading enzymes expression and caused cerebellar neurodegeneration in APP/PS1 transgenic mice. (A) Western blot analysis of NEP and IDE expression in cerebellar neurons treated with conditioned medium. (B) The Aβ_1-40_ ELISA result shows that H-CM induced cerebellar Aβ levels compared with C-CM. (C) Injection of CSF from the lateral ventricle (LV) induced cerebellar Aβ levels compared with CSF from the fourth ventricle (4V) (*P<0.05, ** *P*<0.01). (D) Motor function of APP/PS1 mice treated with H-CM was impaired in a pole test and (E) balance beam test (n = 6 to 11).

On the other hand, we also found that H-CM, which contains metabolites of hippocampal neurons, significantly reduced the expression of IDE and NEP in cerebellar neurons (Figure 4A). Moreover, injection of concentrated H-CM and CSF from the lateral ventricle to the fourth ventricle of APP/PS1 transgenic mice induced cerebellar Aβ levels (Figure 4B and C) and and astrogliosis (data not shown) compared with injection of C-CM after one week of injection.

Cerebellum is important for coordinated movement and balance. Thus, pole test and balance beam test were performed to measure cerebellar function of these mice after one week of H-CM treatment. The behavioural function of APP/PS1 mice treated with H-CM was markedly impaired in both pole test (Figure 4D) and balance beam test (Figure 4E), indicating disturbed balance and cerebellar dysfunction compared with these mice treated with C-CM. These results demonstrated that metabolites of hippocampal neurons caused cerebellar neurodegeneration in AD mice.

### Discussion

Our findings highlight, for the first time, the role of metabolites of neurons in the pathogenesis of Alzheimer's disease. On the one hand, metabolites of cerebellar neurons could protect against AD, while on the other hand, metabolites of hippocampal neurons facilitate AD pathogenesis.

In detail, metabolites of cerebellar neurons reduce Aβ accumulation by inducing Aβ degrading enzymes. A previous report shows that IDE and NEP levels in cerebellum are significantly higher than those in hippocampus and selectively decreased in hippocampus during aging [15]. Thus, our findings about the induction of IDE and NEP levels by treatment with C-CM indicate that the metabolites of neurons may attribute to the difference in IDE and NEP levels between cerebellum and hippocampus. The improvements in cognitive function by metabolites of cerebellar neurons could not be attributed to nonspecific effects on cognition because neither C-CM nor CSF-4V had an effect on the performance of non-transgenic mice (data not shown). The improvement in performance could also not be attributed to nutritional or caloric effects because body weight, activity and coat condition were not different between the groups. Thus, the reversal of Alzheimer's disease-like phenotypes is a result of the significant reduction in brain Aβ levels in APP/PS1 mice treated with metabolites of cerebellar neurons. The levels of Aβ are determined by the balance between its generation and clearance [16], [17]. Aβ is generated from Aβ precursor protein (APP) through two sequential proteolytic cleavages by β-secretase and γ-secretase [18] and is cleared either through receptor-mediated efflux across the blood-brain barrier (BBB) [19], [20] or via peptidolytic mechanisms [21]. Metabolites of cerebellar neurons did not accelerate the clearance of Aβ across the BBB because the levels of peripheral plasma Aβ were remained unchanged (Table S2 and Fig. S1). It is also not possible that metabolites may have contained Aβ degrading enzymes because incubation with C-CM alone did not lead to Aβ clearance (Fig. S2). So, metabolites of cerebellar neurons protect against AD pathogenesis by reducing Aβ accumulation via Aβ degrading enzymes.

According to recent evidence, the expression of NEP and IDE are up-regulated by treatment with ciliary neurotrophic factor (CNTF) [22] and IL4 [23]. IDE is also induced by insulin signal pathway through phosphatidylinoesitol 3-kinase [24]. Thus, the metabolites of cerebellar neurons could contain some neurotrophic factors or small molecules which could activate insulin pathway.

We also investigate the molecular identity of the metabolites derived from the conditioned medium. Firstly, the conditioned medium was separated to two parts by ultra filter. One contains molecules larger than 5 kD (Heavy) and the other are composed of molecules smaller than 5 kD (Light). Then, neurons were treated with these two parts of conditioned medium respectively and according to the Western blot results, the Heavy part of conditioned medium showed little effect on IDE and NEP expression. Conversely, the Light part markedly repeated the effect of total conditioned medium.

Next, we focused on the Light part of conditioned medium and extracted the hydrophobic composition by organic extraction and treated neurons with the hydrophobic and hydrophilic composition respectively. While the hydrophilic part showed little function on IDE and NEP expression, the hydrophobic part completely repeated the effect of total conditioned medium. Based on these results, it is suggested that the active ingredient should be hydrophobic molecule(s) with molecular weight less than 5 kD. Further analysis of these compounds is currently being conducted.

Conversely, metabolites of hippocampal neurons significantly reduced the expression of Aβ degrading enzymes and induced cerebellar Aβ levels. Finally, metabolites of hippocampal neurons caused cerebellar neurodegeneration in APP/PS1 transgenic mice.

It is known that the cerebrospinal fluid (CSF) was primarily generated and secreted by the choroid plexuses (CP) and approximately 10–20% was arised from the brain interstitial fluid, which contains metabolites of neurons [25]. The circulation of CSF is one-direction. CSF flows from the lateral ventricles via the foramina of Monro into the third ventricle, and then the fourth ventricle via the cerebral aqueduct in the brainstem. The location of the choroid plexuses in the lateral, third, and fourth ventricles allows for the possibility of new components being added to the fluid at these points [26]. There is also active exchange of substances between the CSF and its surrounding neurons, for example, CSF-contacting neurons which are located periventricularly or inside the brain ventricles. Thus, after a series of substances exchange, the components of the CSF from lateral ventricle (LV) and the fourth ventricle (4V) maybe different. Moreover, previous evidence show that Na levels in the fourth ventricle are statistically different from those in lateral ventricle [27]. Taken together, the findings about the effect of 4V-CSF on the Aβ levels of APP/PS1 transgenic mice and their learning and memory function could reflect the function of *in vivo* neuronal metabolites.As we all known, the level of Aβ is determined by the balance between its generation and turnover [16]. In familial Alzheimer's disease (FAD), Aβ accumulation is caused by misprocessing of amyloid precursor protein (APP) [28]. However, in the great majority of AD, the sporadic late-onset AD (LOAD), it is decreased Aβ clearance that contributes to the pathological changes of the disease [29]. According to our study, the role of metabolites of neurons in AD pathogenesis is closely associated with Aβ degradation, thus, could be beneficial to the clinical therapy for AD patients.

## Materials and Methods

### Animals

All animal experiments were conducted according to the guidelines established by the NIH Guide for the Care and Use of Laboratory Animals. APP/PS1 transgenic mice of C57BL/6J background and wild-type littermates, were purchased from the Chinese Academy of Medical Science. Cerebrospinal fluid was extracted and collected from the lateral ventricle and fourth ventricles of wild-type C57BL/6J mice with a brain solid positioner placed consistent with the brain atlas. Conditioned medium and CSF was injected into the lateral ventricle of APP/PS1 transgenic mice and wild-type littermates (n = 6 to 10 mice in each treatment group) with a brain solid positioner. 12 month old APP/PS1 transgenic mice were used for the conditioned medium injection; both 4 month old and 12 month old APP/PS1 transgenic mice were used for the CSF injection. Seven days after injection, mice were tested for learning and memory abilities and were then sacrificed for tissue collection.

### Behavioral tests and data analysis

In all behavioral tests, the experimenter was ‘blind’ to the groups to which the individual mice belonged [30].

The detailed method for the Morris water maze has been described previously [31]. Training was performed four times per day for 5 days. One day after the final training, the platform was removed from the pool and each mouse received one 60 s test [32]. The swimming trace was recorded and analyzed for each mouse. The passive avoidance test has been described in detail [33]. Mice were placed one at a time into the illuminated side of a two-compartment apparatus. Upon entering the dark compartment they received a mild electric shock which continued until they returned to the light compartment up to a maximum of 5 s. The retention test took place 24 h after training with the shocker switched off and a maximum latency of 300 s [34].

The detailed method for the pole test has been described previously [31]. The mouse was placed head upward on the top of a vertical iron rough-surfaced pole (diameter 1 cm, height 50 cm). The times taken to turn completely downwards and then to descend to the platform were recorded (with the cut-off limit of 5 min).

In balance beam test, mice were placed on a wooden beam (diameter 1.5 cm, length 40 cm). The beam was elevated 50 cm from the ground and was ended near a home cage. The latencies of mice to get into the home cage were recorded.

### Analysis of Aβ burden in brain

Hemi-brains were removed from mice brains and fixed in acetone. Then hemispheres were cut using a freezing microtome. Aβ plaque burden was detected using thioflavin-S [35] staining or a polyclonal Aβ antibody (diluted 1∶100, Cell signaling) and the digital image was analyzed using Image J software (NIH).

### Plasma and cerebral Aβ content

We homogenized the hippocampus and cerebral cortex in TBS with the protease inhibitors. Homogenates were centrifuged and supernatants from the TBS extraction were stored as soluble Aβ while the pellets were resuspended in a 5 mol/L guanidine HCl and 50 mol/L Tris-HCl (pH 8.0) buffer and stored as insoluble Aβ. Samples were aliquoted and stored at −80°C and analyzed for Aβ40 and Aβ42 using an ELISA kit (USCN Life), according to the manufacturer's protocol. Briefly, protein samples and the detector antibody were incubated with the first Aβ antibody pre-coated on plates for 3 hours at room temperature. After washing, secondary antibody was incubated for 30 minutes. Finally, colorimetric reaction was conducted and absorbance on a spectrophotometer (Bio-Rad Laboratories) at 450 nm was recorded.

### Quantification of gliosis

Five randomly selected, evenly spaced sagittal sections were collected from acetone-fixed and frozen hemispheres of treated and control mice. A polyclonal glial fibrillary acid protein antibody (diluted 1∶100, Santa Cruz Biotechnology) was used to immunolabel astrocytes and a polyclonal CD68 antibody (diluted 1∶100, Santa Cruz Biotechnology) was used to detect microglia. Digital images were captured using a digital camera mounted to a microscope. Images were analyzed using Image-Pro Plus 5.0 software.

### Quantification of synaptophysin

Immunohistochemical staining for synaptophysin was performed on three evenly spaced sections of acetone-fixed treated and control mice. A polyclonal synaptophysin antibody (diluted 1∶100, Santa Cruz Biotechnology)was used to immunolabel the synaptophysin. Digital images were captured and analyzed as described above.

### Primary neuronal culture

The hippocampus, cerebral cortex and cerebellum were dissected from brains of embryonic day 18 (E18) Sprague Dawley rats and digested with 0.25% trypsin to obtain dissociated cells. The resultant cells were cultured in Neurobasal medium (NBM) supplemented with B27 at 37°C in 5% CO_2_. Glial cells were not observed by microscopy in the neuronal cultures at the time of the experiment.

### Preparation of conditioned medium

Primary neurons after 7 to 10 DIC (days of *in vitro* culture) were washed three times with B27-free Neurobasal medium and incubated with B27-free Neurobasal medium for 48 hr. The medium was collected and centrifuged at 800 g (4°C) to remove cellular debris, then aliquoted and stored at −80°C. For injection to APP/PS1 transgenic mice, the conditioned medium was pre-concentrated 30-fold (from 45 ml to 1.5 ml) using a Centricon 100 concentrator device (Christ, German) and then aliquoted and stored at −80°C.

### Real-time PCR

Brain tissues were homogenized and total RNA was isolated using Trizol (Invitrogen). The primers for APP, BACE1, PS1, NEP and IDE SYBR Green PCR were purchased from Invitrogen. Real-Time PCR was performed by Mx3000P (Stratagene).

### Western immunoblotting

Samples were resolved by SDS-PAGE and transferred to a PVDF membrane. The membrane was blocked for 1.5 h in 5% nonfat milk and incubated with primary antibodies for Aβ (polyclonal antibody, diluted 1∶200, Sigma), NEP (polyclonal antibody, diluted 1∶500, Millipore), IDE (monoclonal antibody, diluted 1∶2000, Abcam) and actin (polyclonal antibody, diluted 1∶500, Santa Cruz Biotechnology). The membranes were then incubated with horseradish peroxidase-conjugated anti-mouse IgG or anti-rabbit IgG. Finally, the membranes were developed by a Kodak medical X-ray processor (Kodak).

### Chemicals and statistical analyses

Human Aβ _1-40_ and Thioflavin-S were purchased from Sigma. Data are presented as group means±SEM. A value of *P*<0.05 was considered significant. One-way analysis of variance (ANOVA) was used to evaluate the differences between the experimental groups.

## Supporting Information

Table S1 (0.07 MB DOC)

Table S2 (0.03 MB DOC)

Figure S1 (0.06 MB DOC)

Figure S2 (0.05 MB DOC)
