# Supplementary figures and images for: Metabolites of Cerebellar Neurons and Hippocampal Neurons Play Opposite Roles in Pathogenesis of Alzheimer's Disease
Source: PLoS One. 2009 May 13;4(5):e5530. doi: 10.1371/journal.pone.0005530 (PMC2677455; doi:10.1371/journal.pone.0005530)

**Table S1**

**Metabolites of cerebellar neurons decreases Aβ levels in cerebral cortex**


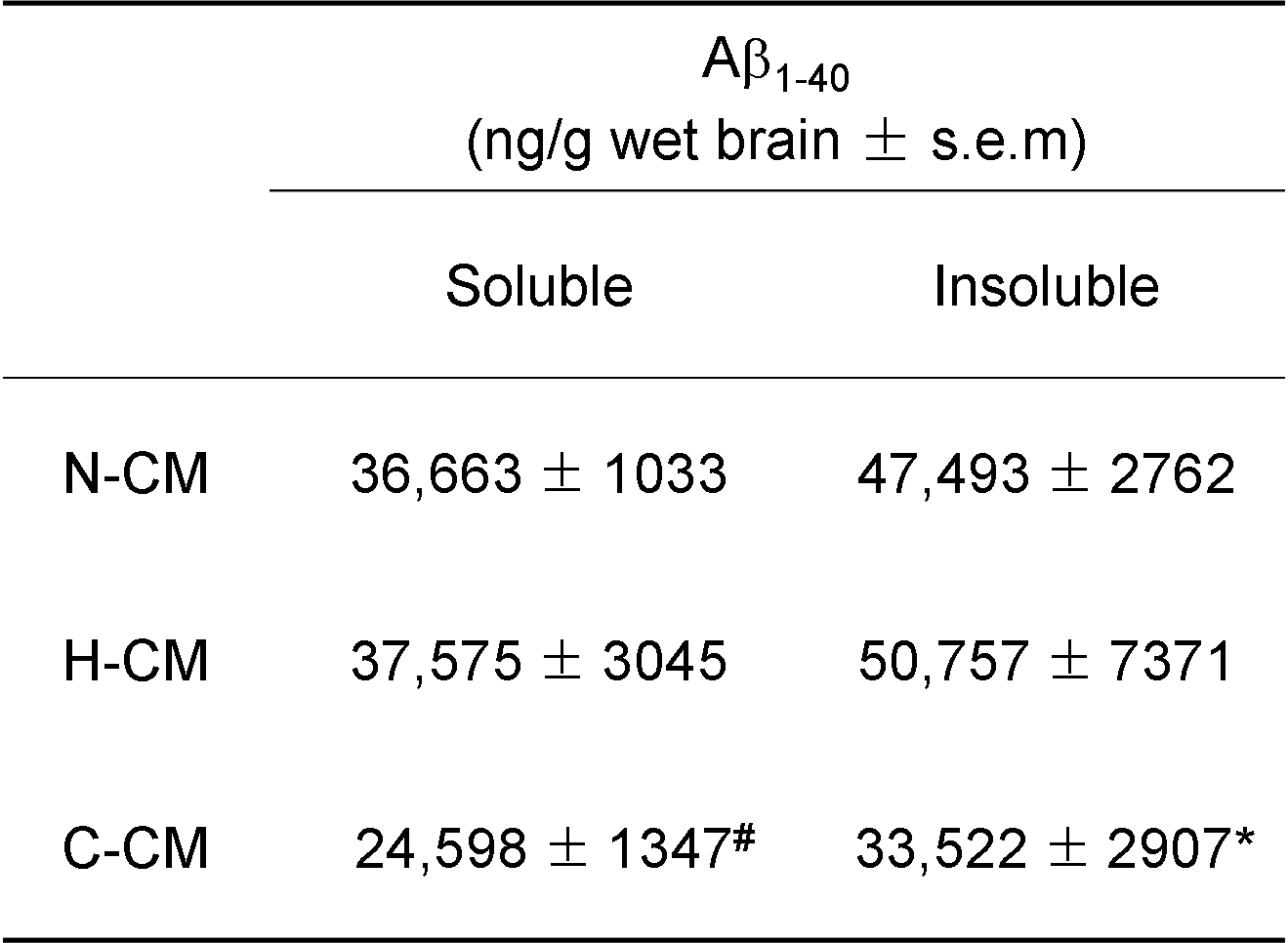


#*P* < 0.001, **P* < 0.05

Supplement: Table S1 — (0.07 MB DOC) [file pone.0005530.s001.doc]

**Table S2**

**Metabolites of cerebellar neurons does not alter peripheral plasma Aβ levels**


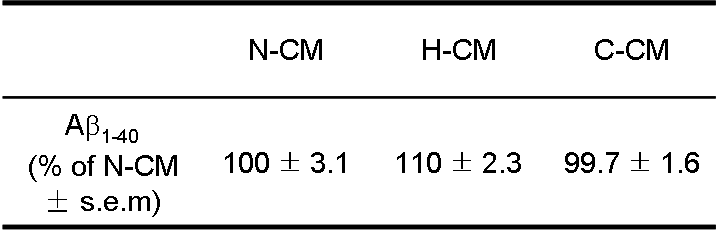

Supplement: Table S2 — (0.03 MB DOC) [file pone.0005530.s002.doc]
