# Supplementary material for: Metabolites of Cerebellar Neurons and Hippocampal Neurons Play Opposite Roles in Pathogenesis of Alzheimer's Disease
Source: PLoS One. 2009 May 13;4(5):e5530. doi: 10.1371/journal.pone.0005530 (PMC2677455; doi:10.1371/journal.pone.0005530)
Supplement: Figure S1 — (0.06 MB DOC) [file pone.0005530.s003.doc]

**Figure S1**

**Metabolites of cerebellar neurons does not alter peripheral plasma Aβ levels.** Peripheral plasma Aβ was extracted and detected by ELISA.

**
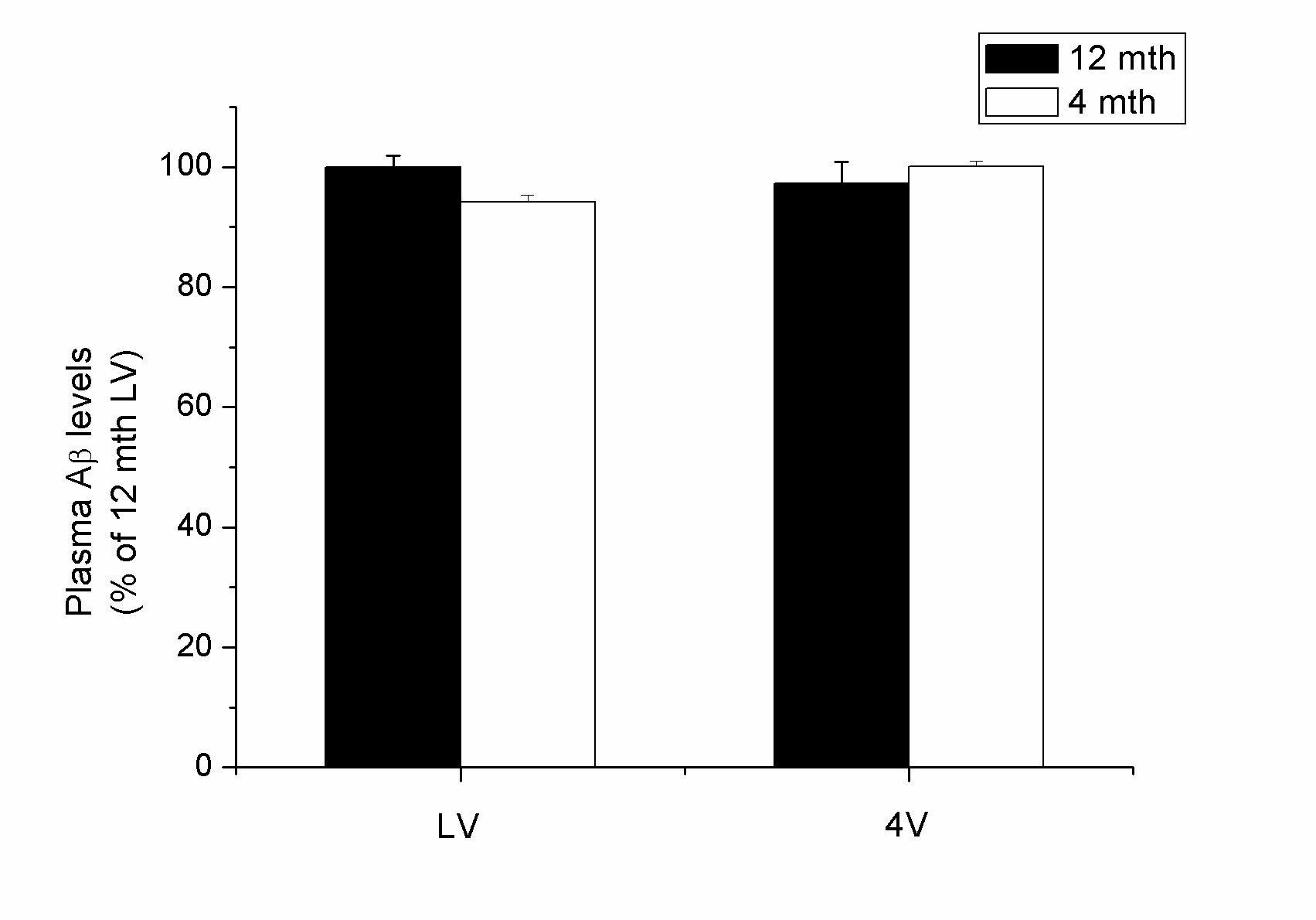
**
