# Supplementary material for: Metabolites of Cerebellar Neurons and Hippocampal Neurons Play Opposite Roles in Pathogenesis of Alzheimer's Disease
Source: PLoS One. 2009 May 13;4(5):e5530. doi: 10.1371/journal.pone.0005530 (PMC2677455; doi:10.1371/journal.pone.0005530)

**Figure S2**

**Metabolites of cerebellar neurons alone did not lead to Aβ clearance.** The Aβ_1-40_ ELISA result shows metabolites of cerebellar neurons alone did not lead to Aβ clearance.


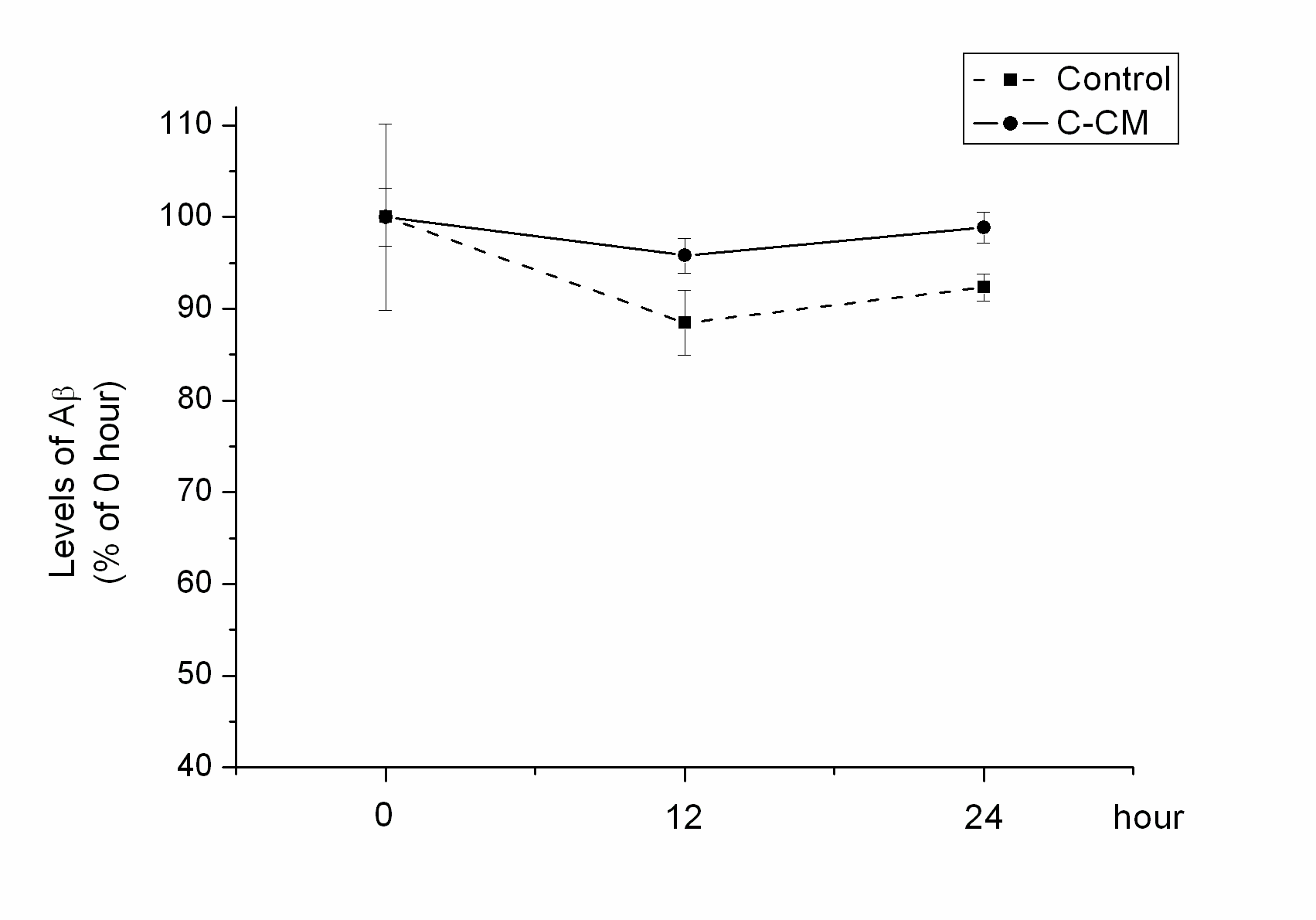

Supplement: Figure S2 — (0.05 MB DOC) [file pone.0005530.s004.doc]
